# Impact of the COVID-19 pandemic on inbound air travel to Canada

**DOI:** 10.14745/ccdr.v50i34a04

**Published:** 2024-04-30

**Authors:** Vanessa Gabriele-Rivet, Erin Rees, Afnan Rahman, Rachael M Milwid

**Affiliations:** 1 National Microbiology Laboratory, Public Health Agency of Canada, St-Hyacinthe, QC

**Keywords:** travel volume, commercial air traffic, IATA, SARIMA, interrupted time series analysis, travel restrictions, Canada, COVID-19

## Abstract

**Background:**

Commercial air travel can result in global dispersal of infectious diseases. During the coronavirus disease 2019 (COVID-19) pandemic, many countries implemented border measures, including restrictions on air travel, to reduce the importation risk of COVID-19. In the context of inbound air travel to Canada, this study aimed to: 1) characterize travel trends before and during the pandemic, and 2) statistically assess the association between travel volumes and travel restrictions during the pandemic.

**Methods:**

Monthly commercial air travel volume data from March 2017 to February 2023 were obtained from the International Air Transport Association (IATA). National and airport-level travel trends to Canada were characterized by inbound travel volumes, the number of countries contributing travellers and the ranking of the top ten countries contributing travellers across the study period, by six year-length subperiod groupings (three pre-pandemic and three pandemic). Using seasonal autoregressive integrated moving average (SARIMA) models, interrupted time series (ITS) analyses assessed the association between major travel restrictions and travel volumes by including variables to represent changes to the level and slope of the time series.

**Results:**

The pre-pandemic inbound travel volume increased by 3% to 7% between consecutive subperiods, with three seasonal peaks (July–August, December–January, March). At the onset of the pandemic, travel volume decreased by 90%, with the number of contributing countries declining from approximately 200 to 140, followed by a slow recovery in volume and seasonality. A disruption in the ranking of countries that contributed travellers was also noticeable during the pandemic. Results from the ITS analysis aligned with the timing of travel restrictions as follows: implementation in March 2020 coincided with a sharp reduction in volumes, while the easing of major restrictions, starting with the authorization of fully vaccinated travellers from the United States to enter Canada in August 2021, coincided with an increase in the slope of travel volumes. Descriptive and statistical results suggest a near-return of pre-pandemic travel patterns by the end of the study period.

**Conclusion:**

Study results suggest resilience in commercial air travel into Canada. Although the COVID-19 pandemic led to a disruption in travel trends, easing of travel restrictions appeared to enable pre-pandemic trends to re-emerge. Understanding trends in air travel volumes, as demonstrated here, can provide information that supports preparedness and response regarding importation risk of infectious pathogens.

## Introduction

Global air travel volumes and interconnectivity increased between 2010 and 2019 ((1)) and, prior to the coronavirus disease 2019 (COVID-19) pandemic, were expected to continue growing ((2)). While higher global connectivity increases international collaboration, trade, and the world’s overall socioeconomic development, it also increases the spread of potential of infectious diseases ((1,3)), such as dengue ((4)), severe acute respiratory syndrome (SARS) ((5)), and influenza ((6)). More recently, the highly transmissible coronavirus, severe acute respiratory syndrome coronavirus 2 (SARS-CoV-2), the causative agent of COVID-19, rapidly spread worldwide following its detection in Wuhan, China at the end of 2019. In response, travel restrictions were implemented by many countries to minimize spread.

On March 21, 2020, the Government of Canada introduced travel restrictions on foreign nationals entering Canada ((7)). Throughout the pandemic, other Canada-wide border measures for travellers coming to Canada were implemented to minimize COVID-19 importation risk, including flight suspensions from selected countries ((8)), pre-departure and on-arrival molecular testing for SARS-CoV-2, and a mandatory 14-day quarantine period for inbound travellers ((9)). Some travellers were exempt from these measures, given their reason for travel, which largely included delivery of essential services, supplies, and equipment ((7)). August 9, 2021, marked the beginning of easing of major travel restrictions with the authorization of non-essential fully vaccinated travellers from the United States to enter the country ((9,10)). The removal of all travel restrictions was completed by October 1, 2022, along with other border measures for testing, quarantine, and isolation ((11)).

In this study, the temporal trends in commercial air travel volumes into Canada from March 2017 to February 2023 were analyzed to gain a further understanding of the impact of the pandemic on inbound travel. The study objectives were to: 1) describe inbound travel patterns both before and during the COVID-19 pandemic, and 2) use an interrupted time series (ITS) analysis to statistically assess the association between inbound travel volumes and the implementation and removal of travel restrictions (as modelled by changes to the level and slope of the time series). The study results have implications for understanding the resilience of the air transportation system under the external stressor of a global pandemic.

## Methods

### Data

Commercial air passenger volume data, aggregated at the monthly level, were acquired from the International Air Transport Association (IATA) for March 2017 to February 2023. The IATA is the trade association for commercial airlines and provides analytics for their air traffic. The data, which are derived from approximately 300 airline companies, represent 83% of global air traffic from 2016 onwards ((12)). The data are presented as the number of passengers on each flight itinerary, which can include one to five stops between the origin and final destination airports. For this study, the IATA data were subset to inbound travel to Canada.

### Descriptive analysis

Inbound air travel to Canada data were summarized at national and airport levels, with the latter consisting of the four largest Canadian airports as the final destination: Toronto Pearson International Airport, Montréal-Pierre Elliot Trudeau International Airport, Vancouver International Airport, and Calgary International Airport. The IATA data were divided into six year-length subperiods beginning in March, to align with the implementation of air travel restrictions. The pre-pandemic subperiods were March 2017 to February 2018 (subperiod −3), March 2018 to February 2019 (subperiod −2), and March 2019 to February 2020 (subperiod −1). The pandemic subperiods were March 2020 to February 2021 (subperiod 1), March 2021 to February 2022 (subperiod 2), and March 2022 to February 2023 (subperiod 3).

Data summaries included the travel volume for each subperiod and the percent change in the passenger volume between consecutive subperiods. To explore seasonal patterns in inbound air traffic, the travel volume and the total number of countries contributing to travel volume were summarized at the monthly level across the six subperiods. Finally, heat maps were generated at the national and airport levels to visually compare the ranking of the top 10 countries contributing to inbound travel during each subperiod. Countries were categorized into one of seven travel volume categories, which were determined by inspecting the distribution of total travel volumes into Canada.

### Statistical analysis

An ITS analysis using seasonal autoregressive integrated moving average (SARIMA) models ((13)) was conducted to evaluate the association between major travel restrictions and inbound monthly travel volumes. For the purpose of this analysis, major travel restrictions are defined as traveller-level measures applicable to the majority of non-essential travellers (e.g., restrictions based on vaccination status). As such, we do not include Notices to Airmen (NOTAMs) in this definition, which, when used during the pandemic, were only applicable to a small proportion of travellers and were therefore not expected to have as big of an impact on travel volume.

Time series data are often serially dependent through time (known as autocorrelation). Seasonal autoregressive integrated moving average models have been used in air travel time series data analysis ((14,15)) as they have the advantage of accounting for seasonality and other autocorrelation. As such, an ITS analysis using the SARIMA modelling approach is robust for assessing the impact of an intervention on the outcome variable compared to the traditional segmented regression ITS, for which the assumption of independent observations is often violated. The model is expressed as SARIMA (p, d, q) × (P, D, Q)_s_ where s refers to the number of observations per season, and parameters p, d, and q refer to the order of the autoregressive process, the degree of differencing, and the order of the moving average process, respectively. Additionally, P, D, and Q represent the analogous terms for the seasonal components.

The premise of an ITS approach is to assess whether the observed values diverge from model-fitted values when accounting for the effect of an intervention. For the present analyses, it was hypothesized that intervention effects from travel restrictions could be modelled by two types of dummy variables—step change and ramp ((13)). Two step changes were used to capture the sharp drop in travel volumes during March and April 2020, respectively. Two step changes were required because travel restrictions implemented on March 21, 2020, only partially impacted the total travel volume that month. A ramp was used to capture rebounding travel volumes starting in August 2021, to coincide with the first instance of easing major travel restrictions, with the authorization of non-essential fully vaccinated travellers from the United States to enter the country. The ITS model was compared with a null hypothesis (H_0_) model that did not include the step and ramp variables. The models were fit using the auto.arima function from the R forecast package to find the best-fitting SARIMA terms accounting for autocorrelation ((16,17)). Residuals from the fitted models were assessed for normality, absence of heteroscedasticity, and autocorrelation using a plot through time, a histogram, an autocorrelation function plot and the Ljung-Box test for autocorrelation. The *p*-values below 0.05 were considered statistically significant for all statistical tests. The ITS and H_0_ models were compared by their fit to the observed data using root mean square error (RMSE) and mean absolute error (MAE) ((18)). All analyses were conducted using the R statistical software environment, version 4.2.1 ((19)).

## Results

**Figure 1:**
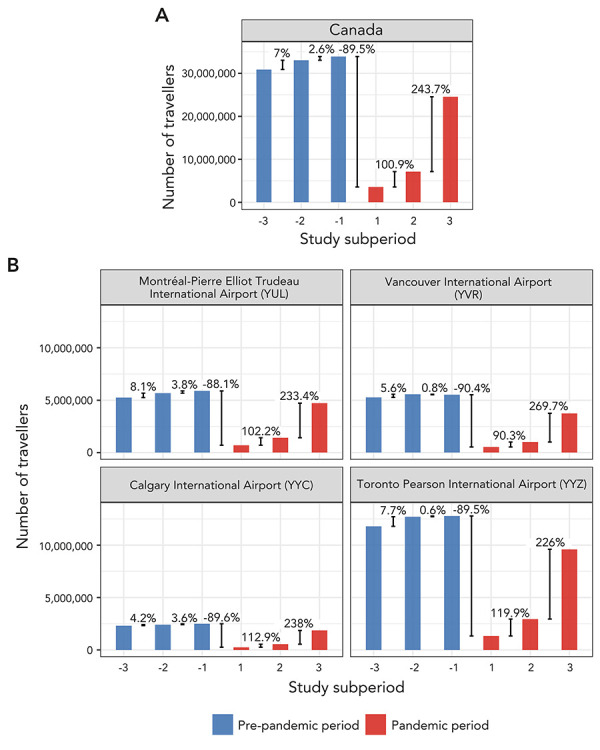
Total incoming travel volumes per study subperiod and percent change between consecutive subperiods for A) Canada and B) each of the four largest Canadian airports as the final destination Note: Subperiods spanned the pre-pandemic (subperiod −3: March 2017–February 2018, subperiod −2: March 2018–February 2019, and subperiod −1: March 2019–February 2020) and pandemic (subperiod 1: March 2020–February 2021, subperiod 2: March 2021–February 2022, and subperiod 3: March 2022–February 2023) study period

Prior to the COVID-19 pandemic, the overall inbound commercial air travel to Canada increased over time with a 7% increase from pre-pandemic subperiod −3 to subperiod −2, and a 3% increase from pre-pandemic subperiod −2 to subperiod −1. By pre-pandemic subperiod −1, there were over 33.9 million travellers arriving in Canada. The onset of the COVID-19 pandemic resulted in a 90% decrease in air traffic volume, with fewer than 4 million travellers entering Canada in pandemic subperiod 1. The travel volume subsequently increased throughout the remainder of the study period (a 101% increase from pandemic subperiod 1 to subperiod 2, and a 244% increase from pandemic subperiod 2 to subperiod 3), allowing a slow recovery to near pre-pandemic levels by pandemic subperiod 3 (24.5 million travellers; **Figure 1**). Similar trends were observed at the airport level, where most travellers (38%–41% per year) landed at Toronto Pearson International Airport, followed by Montréal-Pierre Elliot Trudeau International Airport (17%–20% per year), Vancouver International Airport (14%–17% per year), and, finally, Calgary International Airport (7%–8% per year).

**Figure 2:**
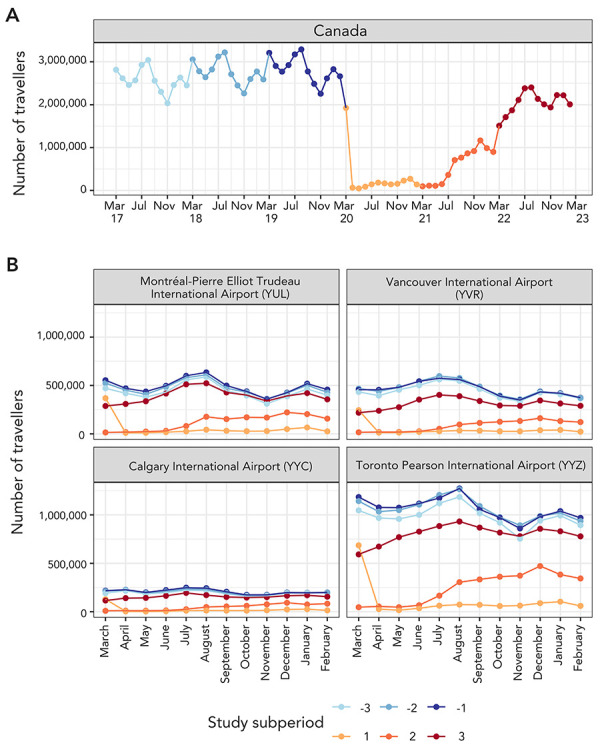
Incoming travel volume to A) Canada and B) each of the four largest Canadian airports as the final destination stratified by month and study subperiod Note: The study period was divided into six subperiods: pre-pandemic (subperiod −3: March 2017–February 2018, subperiod −2: March 2018–February 2019, and subperiod −1: March 2019–February 2020) and pandemic (subperiod 1: March 2020–February 2021, subperiod 2: March 2021–February 2022, and subperiod 3: March 2022–February 2023)

Throughout the pre-pandemic subperiods, the overall and airport-level monthly inbound travel volume was cyclical, with peaks in summer (July–August), winter (December–January), and late winter/early spring (March). Although highly dampened, these trends appear by visual assessment to continue throughout the pandemic, with a rise in travel volume noticeable especially during the summer and the winter months. Similar trends were observed at the airport level (**Figure 2**).

**Figure 3:**
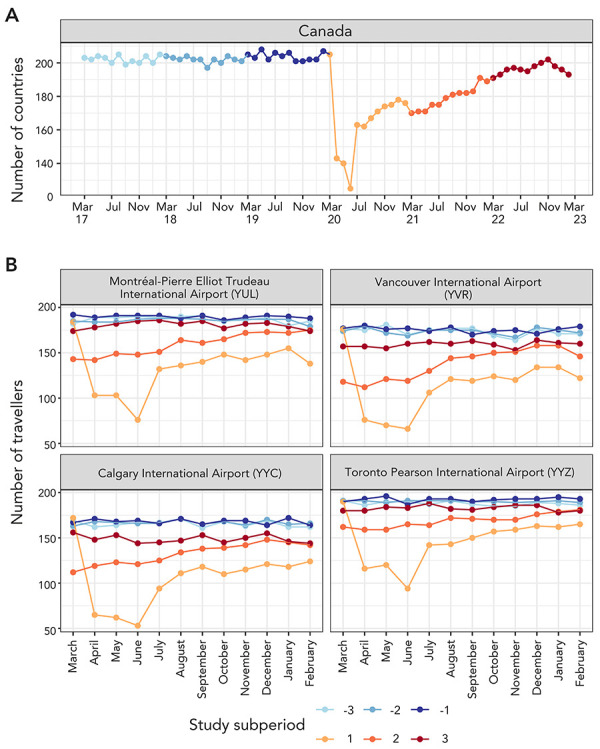
The number of countries with travellers arriving in Canada each month, reported for A) Canada and B) the four largest Canadian airports as the final destination Note: Trends were compared across study subperiods: pre-pandemic (subperiod −3: March 2017–February 2018, subperiod −2: March 2018–February 2019, and subperiod −1: March 2019–February 2020) and pandemic (subperiod 1: March 2020–February 2021, subperiod 2: March 2021–February 2022, and subperiod 3: March 2022–February 2023)

In contrast to travel volume, seasonal patterns were not evident when data were summarized as the number of countries contributing travellers to Canada. Prior to the COVID-19 pandemic, travellers from approximately 200 countries contributed to inbound travel to Canada each month, decreasing to approximately 140 countries in April and approximately 125 countries in June 2020. The number of contributing countries continued to trend upward from June 2020 onward, reaching pre-pandemic levels during pandemic subperiod 3 at the national level and for all airport destinations, except Calgary and Vancouver international airports (**Figure 3**).

**Figure 4:**
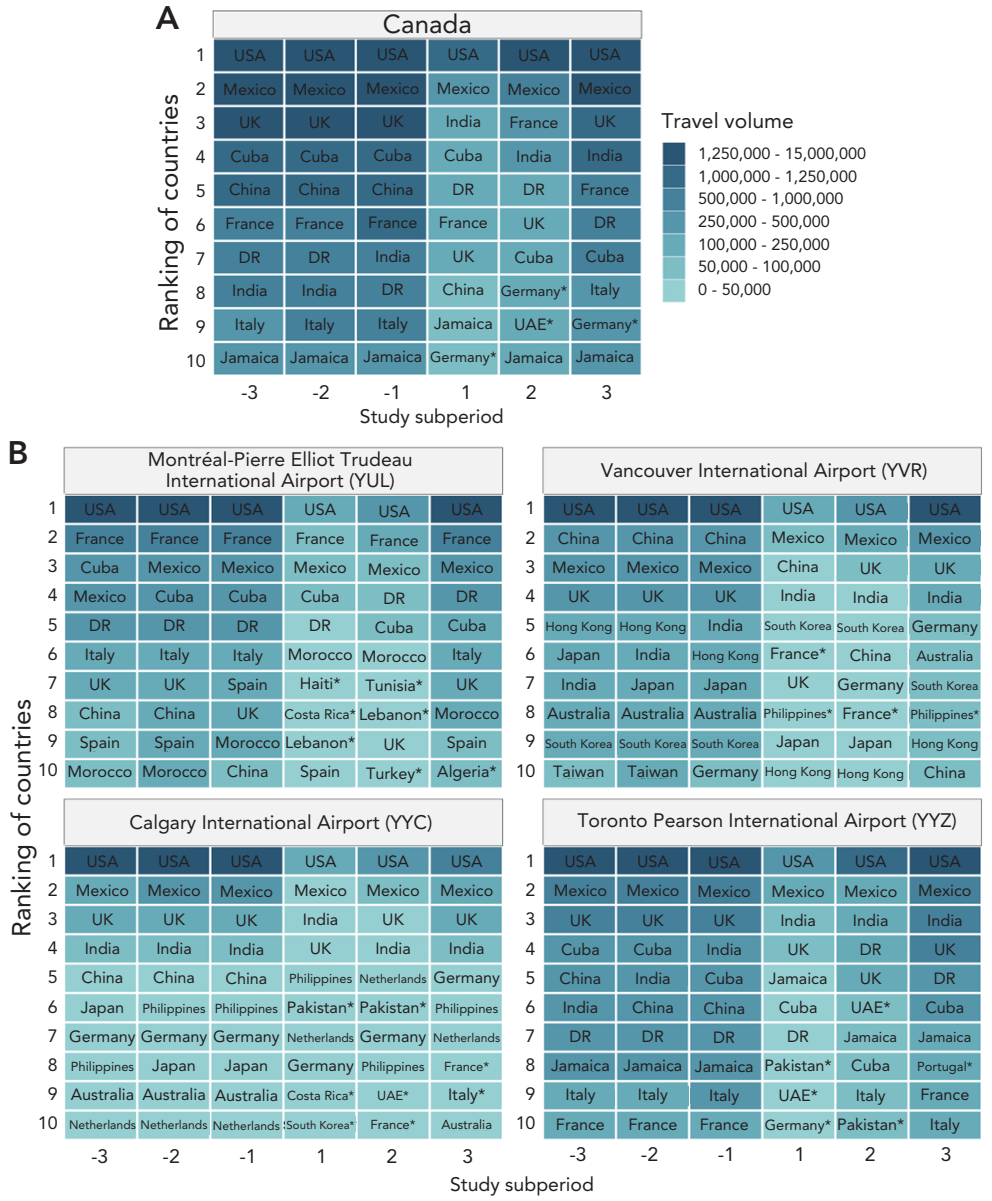
Ranking of incoming travel volume by origin country for incoming travel volume to A) Canada and B) each of the four largest Canadian airports as the final destination Abbreviations: DR, Dominican Republic; UAE, United Arab Emirates; UK, United Kingdom; USA, United States of America Notes: The rankings were stratified by study subperiod (pre-pandemic: subperiod −3: March 2017–February 2018, subperiod −2: March 2018–February 2019, and subperiod −1: March 2019–February 2020; and pandemic: subperiod 1: March 2020–February 2021, subperiod 2: March 2021–February 2022, and subperiod 3: March 2022–February 2023) Asterisks indicate countries that were found within the top ten contributors of incoming travel volume during the pandemic subperiods but not the pre-pandemic subperiods

The United States consistently contributed the majority of air travellers at both the national and airport levels throughout the study period. Before the COVID-19 pandemic, the order of the top ten countries contributing to inbound travel was relatively consistent between subperiods, though the ranking varied between airports. At the onset of the pandemic, there was a large decrease in travel volume per country of origin, as well as a disruption in the ranking of the top ten contributing countries to travel volume at the national and airport levels throughout the pandemic period. For example, some airports had new countries entering the top ten (e.g., United Arab Emirates for the Toronto Pearson International Airport) and some countries moved higher in their rank of contribution (e.g., Netherlands for the Calgary International Airport) (**Figure 4**).

The best fit H_0_ and ITS models were SARIMA (0,1,1) x (1,0,0)_12_ and SARIMA (2,0,0) x (2,1,0)_12_, respectively. In the ITS, variables included to model the impact of the implementation of travel restrictions on inbound air travel volumes to Canada were statistically significant (i.e., two step changes estimating a reduction in travellers for March 2020 and April 2020). Also statistically significant was an increase in the slope of travellers in August 2021 compared with what was expected in the absence of travel restrictions (**Supplemental material**). Both the H_0_ and ITS models passed the Ljung-Box tests (Q*=7.6141, df=12, lag=14, *p*-value=0.815; Q*=7.2782, df=10, lag=14, *p*-value=0.699), however, the additional variables in the ITS model intended to capture changes in travel restrictions resulted in better model performance metrics for RSME (H_0_: 303,353.2, ITS: 124,124.6) and MAE (H_0_: 198,609.5, ITS: 82,132.2). Residual assessments of the H_0_ model suggest that it did not account for the effect of travel restrictions, as expected, and as observed by the stark drop in residuals during March 2020 and April 2020. On the other hand, residuals for the ITS model show that the model does not adequately account for differences in the magnitude of seasonal patterns between the pre-pandemic and pandemic periods. Details for model diagnostics are included in the supplemental material.

## Discussion

This study analyzed temporal trends in inbound commercial air travel volumes into Canada before and during the COVID-19 pandemic and included a statistical assessment of COVID-19 travel restrictions on travel volumes. While the initial disruption was biggest at the onset of the pandemic during the strictest border measures, descriptive and ITS analysis results show that travel volume and seasonal patterns gradually returned to pre-pandemic trends as travel restrictions were eased. Conversely, the ranking of countries by incoming volume did not return to pre-pandemic levels to the same extent as the other measures.

Using the COVID-19 pandemic as a case study, the present analyses highlight the impact that an international crisis can pose on travel patterns (volume, seasonality, ranking of contributing countries). Air travel has been shown to be an important factor in the dispersion of infectious diseases worldwide ((20)), and such radical changes in air traffic are likely to have direct implications on the importation risk of infectious pathogens into Canada. For instance, the risk of importation depends on the travel volume from high-incidence source countries, as previously reported for COVID-19 and other infectious diseases ((21,22)). Similarly, we would expect that a change in the ranking of countries that contribute travellers would alter the overall importation risk if the countries differed by disease incidence. The present study results have also shown a gradual return of travel volume patterns to pre-pandemic levels as travel restrictions were eased, demonstrating the resilience of inbound air travel in Canada. Thus, during future outbreaks of emerging and re-emerging infectious diseases, if current or projected air travel volume data are unavailable, “business as usual” patterns (i.e., through historical data under usual circumstances) can still be relevant for informing situational awareness and intervention strategies.

The present ITS analysis was a simple approach to assess for the effect of travel restrictions on travel volumes into Canada using basic intervention impact shapes. Results from this analysis suggest that the implementation of the first travel restrictions on the entry of foreign nationals into Canada ((9)) in March 2020, in response to the global increase in cases ((23)), catalyzed the initial downtrend in travel volume observed at that time, as found elsewhere in the world ((24)). Following that, the easing of travel restrictions in Canada in August 2021 coincided with a significant increase in inbound travel volume during the COVID-19 pandemic period. Even though the modelled effects were statistically significant, other factors not included in the model could be associated with observed trends in travel volumes during the pandemic period. For instance, other border measures, such as testing, and quarantine requirements can act as major disincentives for people to travel. Furthermore, NOTAMs that were implemented over short periods to ban travellers from specific countries from entering Canada (e.g., the United Kingdom [December 2020 to January 2021 ((25))], Pakistan [April to June 2021 ((26))], India [April to September 2021 ((27))], Mexico and Caribbean countries [January to April 2021 ((28))], and Morocco [August to October 2021 ((8,9))]) likely contributed to reducing travel volumes transiently. Global travel can also be impacted by complex and interconnected factors related to economic production, trade, and tourism ((29)), as well as people’s willingness to travel given risk perceptions of COVID-19 ((30)). Given the complexity of the air travel system during the pandemic, future research could benefit from exploring more complex models, for example using transfer functions to better capture the observed effect ((13)) or applying alternative methods to adequately adjust for changes in seasonal patterns, as observed during the pandemic ((31)). Furthermore, a future extension of the study could involve investigating the potential impact of air travel restrictions on COVID-19 importation rates to assess for their effectiveness.

## Limitations

There are other limitations within the study data and analysis. First, the IATA data does not include all global air traffic ((12)). Although the majority of travel volume data (83%) was available for the analysis, it is possible that some trends may have been overestimated, underestimated, or missed. Furthermore, it is important to note that the study results are in the context of the COVID-19 experience in Canada. It is intuitive to expect that travel restrictions implemented for future pandemics would cause a decrease in travel volumes, dampening of seasonal patterns, and re-ordering of the ranking of countries that contribute travellers, as demonstrated in this study. The nature of these impacts, however, will depend on the context of the air travel interactions with Canada given trade, personal travel (e.g., tourism, education, visits to family), the epidemiology of the disease, and the potential for the implementation of travel restrictions.

## Conclusion

In this study, a method is presented to help understand how inbound air travel patterns can be impacted by travel restrictions, as demonstrated in the context of the COVID-19 pandemic in Canada. The approach characterizes the behaviour of the system during standard and unusual circumstances, as shown descriptively by trends in travel volume, seasonality and contributions from countries, and statistically as significant impacts in the implementation and removal of disruptions. While study results indicate that interventions implemented in response to the pandemic have the capacity to disrupt inbound travel patterns at both national and arrival airport levels, they also suggest a gradual return to expected travel patterns and, hence, resilience of the air travel system to major disruptions. Frequent monitoring of air travel patterns, during “business as usual” and disruptive global events, can help public health professionals better inform emergency preparedness and response efforts aimed at reducing importation risk. The study opens avenues for future research in the intersecting fields of air transportation and public health.

## Supplemental material

These documents can be accessed on the Supplemental material file.
